# Impact of SARS-CoV-2 infection on the cognitive functioning of patients institutionalized in nursing homes

**DOI:** 10.1186/s12877-024-05210-y

**Published:** 2024-07-17

**Authors:** Bibiana Trevissón-Redondo, Eduardo Pérez-Boal, Cristina Liébana-Presa, María Cristina Martínez-Fernández, Marta Elena Losa-Iglesias, Ricardo Becerro-de-Bengoa-Vallejo, Eva María Martínez-Jiménez

**Affiliations:** 1 Salbis Research Group, Faculty of Health Sciences, University of León, León, Spain; 2 Faculty of Health Sciences, University of León, León, Spain; 3 Faculty of Health Sciences, University Rey Juan Carlos, Madrid, Spain; 4 Faculty of Nursing, Physiotherapy and Podiatry, Complutense University of Madrid, Madrid, Spain

**Keywords:** SARS-COV-2, Mini-mental state, Cognitive impairment, Homes for the aged

## Abstract

**Background:**

COVID-19 disease affected the cognitive level of institutionalized patients in nursing homes, especially in the older subjects regardless of gender. This study aims to assess cognitive impairment using the Mini-Mental State Examination (MMSE) before and after COVID-19 infection, and to determine whether these changes varied based on gender.

**Methods:**

A pre- and post-COVID-19 study was conducted, involving 68 geriatric patients (34 men and 34 women) from two nursing homes. Cognitive impairment was assessed using the MMSE.

**Results:**

COVID-19 infection had a notable impact on the cognitive health of older adults residing in nursing homes, primarily attributed to the social isolation they experienced. This effect was more pronounced in older individuals. A comparison of the MMSE results by gender before and after contracting COVID-19 revealed significant differences in attention and calculation, with women obtaining the worst score before the virus. However, following their recovery from the virus, men demonstrated significantly lower scores in time and space orientation and evocation.

**Conclusion:**

COVID-19 has led to a decline in cognitive functioning, significantly worsening the mental state of older individuals, even after recovery from the virus. Consequently, it is crucial to implement proactive measures to prevent isolation and safeguard the cognitive well-being of this vulnerable population.

## Introduction

As reported by the World Health Organization [1], the global impact of the novel coronavirus (COVID-19) has been significant, with over 775 million infections and more than 7 million deaths worldwide. In the initial stages of the pandemic, in response to the health crisis, Spain declared a state of emergency (Royal Decree 463/2020, March 14) [2] with significant social restrictions.

In a highly contagious disease such as COVID-19, the severity and progression of the disease are determined by host-related factors. The key risk factors for COVID-19 include age, male gender, obesity, smoking, and underlying chronic conditions such as hypertension, type 2 diabetes mellitus, and others [3]. In particular, the evidence indicates the necessity for the collection of sex-disaggregated data, as it provides valuable information on both the biological aspects of an infectious disease and the social and economic factors that influence the risk of exposure in real time [4]. The most common clinical symptoms in individuals with COVID-19 include fever, cough, muscle pain, fatigue, dyspnoea, anosmia, and ageusia [5]. In addition, some cases may present with increased sputum production, headache, hemoptysis, diarrhea, and myalgia [6]. The average recovery time for mild cases is approximately two weeks, while severe cases may require three to six weeks for recovery [7]. Following recovery from COVID-19, rehabilitation becomes crucial, particularly for the elderly population who may have experienced social isolation [8]. Rehabilitation plays a vital role in enhancing mood, alleviating anxiety and dyspnoea, as well as reducing associated complications, improving functionality and quality of life for patients. Following the acute phase of the disease, patients may experience ongoing physical, emotional, and psychological issues, which may result in prolonged disability. This situation can contribute to a complex and multifactorial disability, which requires ongoing care and adequate rehabilitation management [9].

Confinement due to the COVID-19 pandemic can lead to social isolation, which is a risk factor with consequences closely related to health [10] and increases the risk of dementia and cognitive impairment (CI) in older people [11]. Furthermore, in light of the aforementioned social isolation, it is imperative that the rehabilitation process incorporates an assessment of functional impairment. This may include impairments affecting the performance of activities of daily living (ADL) and CI. A scoping review of the literature on older adults has identified an increase in depression, anxiety, and neuropsychiatric symptoms during the pandemic. Furthermore, the review has indicated that restrictions on virus containment and impaired communication may be contributing to cognitive changes and stress-related symptoms [12]. Consequently, the global pandemic had a profound effect on older adults with mild cognitive impairment or dementia, particularly in the domains of communication, mood, movement, and compliance with new medications [13]. Previous studies have investigated the impact of the COVID-19 pandemic on patients with dementia and Alzheimer’s disease [14–18]. However, there has been a paucity of research examining the impact of the pandemic on elderly patients without cognitive impairment. A study has observed changes in brain function three months after the onset of the COVID-19 pandemic, indicating the potential for long-term effects. Among the infected patients, 68.33% exhibited neurological symptoms and 50% of those who recovered continued to experience symptoms three months later [19]. A meta-analysis of the cognitive effects of COVID-19 in previously unimpaired adults revealed a significant difference in Montreal Cognitive Assessment (MoCA) scores between post-COVID-19 patients and controls (mean difference=-0.94, 95% confidence interval [CI] -1.59, -0.29; *P* = 0.0049). This impairment in cognitive functioning was present in the acute phase and six months post-infection [20]. A study conducted in Dutch nursing homes (NH) found that three months post-infection with SARS-CoV-2, residents were fully functioning as before infection. Similarly, the mean cognitive functioning remained unchanged, while average social functioning decreased [21]. Nevertheless, a further study of comparable characteristics revealed that the residents did not exhibit any clinically significant adverse effects on mood, behaviour, cognitive and social functioning as a result of the confinement [22].

Consecuently, in geriatric patients, the evaluating of cognitive status is of significant importance for enhancing care during potential new epidemic outbreaks that may necessitate enforced isolation. Therefore, the aim of this study is to assess cognitive status in nursing home patients before and after suffering COVID-19, using the Mini Mental State Examination (MMSE), to check whether cognitive status worsens after overcoming the infection, which of the dimensions are more affected and whether the gender of the subject influences the results. The research hypothesis is based on the literature mentioned above, which suggests that COVID-19 increases cognitive decline in the elderly population, putting them at risk. These effects are present both during and after the disease, and they negatively impact the health and quality of life of patients.

## Materials and methods

### Study design

Longitudinal pre- and post- COVID-19 disease study. Authorization was requested from the management of the two nursing homes under the study and informed consent was requested from all patients participating in the study. This study was approved by the ethics committee of the University of León (ETICA-ULE-053-2021). The data were collected from March to December 2020. The study was conducted in two nursing homes, one with 60 beds and the other with 30. These centers are managed as a single entity and have comparable patient volumes and care standards. The study involves 68 residents who survived COVID-19 and were eligible for testing due to their sufficient cognitive level to response to the tests. No participants were vaccinated at the point of this study as it was conducted in the early stages of the pandemic and vaccines were not yet available. Moreover, random sampling of subjects was not possible, as the entire sample was affected by COVID-19.

### Sample

The sample size was calculated using the difference between the pre- and post-event Wilcoxon Signed – Rank Test for one sample using the G*Power 3.1.9.2 software. A two-tailed hypothesis, an effect size of 0.50, an α error probability of 0.05, with a β level of 20% and a desired power analysis of 80% (1-β error probability) were used to calculate the sample size. As a result, a total sample size of 57 participants was calculated. Samples were recruited by a consecutive sampling method using a successive and non-aleatorized simple method.

### Variables and measurements

Cognitive status in nursing homes is regularly assessed using the MMSE. The MMSE is a quick and simple measure that assesses seven domains of cognitive functioning and has been shown to have both good test-retest reliability (0.80–0.95) [23]. The score determines the normality or degree of impairment that the resident may be experiencing. If the resident cannot answer a question for a reason not related to mental illness (e.g. the patient cannot read and is asked to do so), the question should be eliminated and a ratio proportional to the maximum possible score should be obtained [24].

The MMSE evaluates various cognitive functions, such as orientation (autopsychic, in time and place), short and long-term memory (fixation and delayed recall), attention, language (verbal and written comprehension, verbal expression repetition and articulation and written expression), praxis (by written and verbal command) and visuo-constructive ability [24]. Previous meta-analyses indicate that the MMSE should primarily serve as a screening test rather than a tool for case detection [25]. Meta-analyses have found that in non-specialist settings (e.g. nursing home) the sensitivity, specificity, positive predictive value and negative predictive value were 82.1%, 86.1%, 40.5% and 97.7% respectively [26].

### Data collection

In old people’s homes in Spain, particularly in Castilla y León, where the study was conducted, the cognitive status of the residents was assessed to determine their degree of dependency. This was a parameter established by Royal Decree 504/2007 of April 20, which set an assessment scale for determining the dependency situation established by the Law 39/2006, of 14 December. This decree deals with the promotion of personal autonomy and care for people in a situation of dependency. Therefore, based on this regional law, the MMSE has been successfully used in numerous studies and is still widely used today as a simple method to assess cognitive status [17]. Measurements of the MMSE indices were taken no more than 3 months before the illness and were repeated after discharge by the medical team no more than 3 months after overcoming the infection. The following socio-demographic variables were analyzed from the information in the medical records: age, gender, height and weight.

### Participants eligibility

The study included patients aged 65 and above who were residents of the nursing home and had been infected with COVID-19. These patients were required to demonstrate an adequate cognitive level to understand and respond to the questionnaire. Patients with dementia were not excluded. Exclusion criteria were refusal to give informed consent and inability to understand and carry out study instructions, and absence of COVID-19 infection. Table 1 presents the clinical characteristics of the sample in terms of history.

**Table 1:** Clinical characteristics of the patients living in the nursing home

	Female *n* = 34(100%)	Male *n* = 34(100%)
Cardiovascular disease	25 (73,5%)	30 (88,2%)
Diabetes	29(85,3%)	21(61,8%)
Dyslipidemia	24 (70,6%)	25(73,5%)
Insomnia/anxiety	22 (64,7%)	19(55,9%)
Thyroid pathology	19(55,9%)	7(20,6%)
Respiratory disease	3(8,8%)	5(14,7%)
Tumor	3(8,8%)	2(5,9%)
Other*	4(11,8%)	7(20,6%)

*Note Osteoarthritis or arthritis, Parkinson’s disease, depression, lymphomas, and HIV

### Setting

The MMSE was evaluated for up to three months prior to COVID-19 infection and three months post-infection, permitting an assessment of the cognitive state resulting from COVID-19 infection.

Evaluation of elderly participants followed the health authorities’ guidelines. The virus was detected via polymerase chain reaction (PCR) and antigen tests to facilitate patient transfer to the “clean zone” in the residences where patients were isolated and segregated into clean or dirty zones based on the disease status. Patients were re-evaluated for MMSE following negative antigen detection and transferred to the clean zone. It is noteworthy that patients who recovered from COVID-19 showed improvement in their condition and were able to resume their previous lifestyle, despite facing some restrictions.

The nursing homes were separated into areas according to floors, enabling residents to move about on their floor. The dining and living areas were expanded to allow elderly individuals to engage in their regular activities. However, it is worth noting that during the most severe days of the outbreak, as with other infections, elderly patients were confined to their beds.

The functioning of the facilities was compelled to follow emergency protocols, leading to the suspension of many prior activities, and altering the daily routine of the elderly. However, this does not account for the significant decline in cognitive function withing a relatively brief period.

### Statistical analysis

A descriptive analysis of participants’ characteristics was performed. Normal data distribution was evaluated using the Shapiro-Wilk test; the result indicated that the data did not follow a normal distribution. Descriptive data are presented as percentages, medians and interquartile ranges. Wilcoxon signed-rank tests were employed to assess the disparity in MMSE scores prior to and post infection with the coronavirus. The Mann-Whitney U statistic was employed to ascertain differences in the genders of the participants. IBM SPSS v27.0 was used for data analysis.

## Results

The total number of participants was 68, 34 males and 34 females, sociodemographic data are shown in Table 2, where it can be seen that there are no statistically significant differences for the groups except for height. Primary education was the most common level of education among residents, representing 75%. The majority of the 68 residents received regular visits from family members; only 10.29% of them did not receive regular visits. Regarding the level of education, it was quite homogeneous between men and women, of the total sample, 75% had primary education, 19.11% had secondary education and only 5.88% had received university education. If we take the perception of state aid to establish the economic level, 60.29% of residents received some type of financial aid, twice as many women receive aid compared to men.

Regarding the patients’ clinical history, there were no individuals with psychiatric pathology in the residences. However, there were individuals with varying degrees of cognitive deficit or dementia, who are monitored using the MMSE. Chronic diseases identified in the total sample are included in Table 1. Out of the total sample, only 2.94% did not have any chronic diseases. This percentage corresponds to 5.88% of the women, as all the men had at least one of the chronic diseases mentioned above.

**Table 2:** Sociodemographic variables and descriptive data of the sample population according to gender

Demographic and descriptive data	Total group (*n* = 68)	Male group (*n* = 34)	Female group (*n* = 34)	*p*-value
	Median (IQR)	Median (IQR)	Median (IQR)	
Age (years)	86.00 (8.00)	82.50 (7.25)	87.50 (10.25)	0.060
Weight (Kg)	68.00 (22.75)	71.50 (19.25)	68.00 (24.00)	0.224
Height (cm)	169.50 (15.75)	175.50 (14.00)	163.50 (12.00)	< 0.001
BMI (Kg/m2)	23.68 (7.08)	22.74 (4.71)	24.68 (8.10)	0.348

*Abbreviations* BMI, body mass index; IQR: interquartile range U Mann Whitney *p* < 0.05 was considered statistically significant

Table 3 shows the results of the MMSE, before and after infection with COVID-19. All items and the overall score show significant differences. As can be seen, each of the individual dimensions of the MMSE and total score have been significantly reduced after COVID-19 infection.

**Table 3:** Pre and post COVID-19 MMSE results

Variables	Before COVID-19 (*n* = 68) Median (IQR)	After COVID-19 (*n* = 68) Median (IQR)	*p*-value	Z Value
Orientation in time	4.50 (1.00)	3.00 (2.75)	< 0.001	-6.505
Orientation in place	4.00 (2.00)	2.00 (2.00)	< 0.001	-6.682
Memory	3.00 (1.00)	1.00 (1.75)	< 0.001	-5.847
Attention and Calculation	4.00 (1.00)	2.00 (4.00)	< 0.001	-5.752
Evocation	3.00 (1.00)	1.00 (1.00)	< 0.001	-5.125
Nomination	7.50 (2.00)	5.00 (4.00)	< 0.001	-6.533
Total score	26.0 (6.75)	15.00 (14.75)	< 0.001	-7.119

*Abbreviations* IQR: interquartile range; U Mann Whitney *p* < 0.05 was considered statistically significant

Table 4 compares the MMSE results by gender before and after suffering the COVID-19 infection. In the case of women, the attention and calculation item show a significant decrease in their score before the disease. For men, the dimensions of orientation in time, orientation in place and evocation have shown a significant decline after COVID-19.

**Table 4:** Pre and post COVID-19 results of MMSE by gender

	Before COVID-19 (*n* = 68) Median (IQR)			After COVID-19 (*n* = 68) Median (IQR)		
Variables	Female (*n* = 34)	Male (*n* = 34)	p-value	Female (*n* = 34)	Male (*n* = 34)	p-value
Orientation in time	4.00 (2.00)	5.00 (1.00)	0.385	3.00 (2.00)	2.00 (2.25)	0.050
Orientation in place	4.00 (2.00)	4.00 (2.00)	0.458	3.00 (3.00)	1.50 (3.00)	0.006
Memory	3.00 (1.00)	3.00 (1.00)	0.801	2.00 (1.25)	1.00 (2.00)	0.066
Attention and Calculation	3.00 (1.25)	4.00 (1.25)	< 0.001	2.00 (3.25)	1.50 (4.00)	0.361
Evocation	2.00 (1.00)	3.00 (1.00)	0.362	2.00 (2.00)	1.00 (2.00)	0.032
Nomination	7.00 (3.00)	8.00 (1.25)	0.067	5.00 (4.00)	3.50 (4.25)	0.177
Total score	25.00 (8.50)	26.00 (6.00)	0.068	16.50 (14.25)	10.50 (15.75)	0.075

*Abbreviations* IQR: Interquartile range. U Mann Whitney test was used *p* < 0.05 was considered statistically significant

## Discussion

In this study, the cognitive status of patients who contracted SARS-CoV-2 was evaluated using the MMSE. The MMSE was employed to measure various cognitive functions, including orientation, short and long-term memory, attention, language, praxis, and visuo-constructive ability [24]. The MMSE has been used to assess CI resulting from dementia [18], social isolation [10] and the mental deterioration suffered by age [17].

Social isolation resulting from confinement, quarantines established after COVID-19 infections and prolonged stays in the ICU can negatively impact the quality of life and cognitive abilities of older patients [18]. Our research indicates that the cognitive faculties of elderly individuals in long-term care settings, who contracted COVID-19 and underwent isolation or hospitalization, suffered impairment following recovery. A study conducted in a NH in northwest Spain, with comparable characteristics to those observed in our study, indicates that cognitive and functional scores were lower, and depression scores were higher, following strict blocking [27]. A systematic review of the impact of the COVID-19 pandemic on individuals with cognitive impairment living in residential care homes reveals a negative impact of the virus on these individuals. Furthermore, the study indicates that social restrictions may have contributed to cognitive impairment in all residents, which is also associated with mental health and behavioural problems [28].

The main risk factors for COVID-19 disease are gender and age [29]. A study found that infection was more severe for males than females within similar age groups [30], and men a higher mortality rate [31]. These findings could clarify why men, despite having a lower mean age, exhibited worse outcomes in evaluation of activities of daily living after experiencing COVID-19 than women. However, the results indicate that women have lower scores after suffering the infection, suggesting aging as a crucial factor for deterioration [32]. Additionally, the average age of women in this sample is 87.72 years, which is higher than that of men. Research indicates that mortality rates reach above 95% at the age of 80 years, which is the most significant [33].

While there are studies describing the effects of the COVID-19 emergency on elderly institutionalized populations, few investigations have examined the relationship between cognitive sequelae resulting from COVID-19 and the MMSE. The MMSE, a commonly used tool for assessing CI, has shown remarkable correlations with COVID-19-related sequelae suffered by institutionalized patients. By using the MMSE, cognitive impairment can be quantified, classified, and potentially prevented following the resolution of COVID-19. Several studies have used the MMSE to detect cognitive impairment due to COVID-19 in older adults [14–16]. One study reveals how the MMSE has been used to look at the effects of the pandemic in older adults with dementia or mild CI who live alone. In this case, no individuals were infected by the disease, and their activities were not diminished by the COVID-19 pandemic, just as there were no differences in MMSE scores. This may be due to the lower perceived risk of contracting the disease [15]. Conversely, a study conducted at a long-term residential centre in the Netherlands found that there were no adverse clinical effects resulting from isolation during the pandemic. This could be attributed to the efforts of healthcare personnel, as demonstrated by the Dutch study, and the imposition of restrictive isolation measures in each country, which resulted in a high level of isolation in Spain with minimal interaction between residents and healthcare personnel [22]. In contrast, another study reports a 17.5% reduction in the ability to perform activities of daily living and a 17.4% worsening of cognitive impairment in the recovery of institutionalized residents who overcame infection, three months after infection [21].

However, another study suggests that individuals with CI and Alzheimer’s disease (AD) experienced a decline in scores on the MMSE and MoCA. It is possible that the COVID-19 pandemic may have contributed to this decline [16]. Patients with mild CI, dementia with Lewy bodies (DLB) and AD found that isolation and physical inactivity were associated with an acceleration of cognitive decline, as measured by the MMSE and MoCA, over a one-year follow-up period. Specifically, in patients with AD and mild CI, MMSE scores were significantly lower after the follow-up, with a decrease of approximately 3 points in final values. Moreover, a positive correlation has been found between reduced physical activity, sleep disturbances, and a decline in MMSE scores in patients with AD [14].

The limitations of this study include the following: Firstly, the small sample size is not representative of the overall population, hence the results should be interpreted with caution. The limitations of the instrument for determining CI must be highlighted. To increase accuracy, it should be combined with other types of neurological assessments. In addition, there is a geographical restriction on conducting this study, rendering the findings non generalizable. On the other hand, it should be noted that this study could only be carried out on valid people able to answer the questions, in addition to the high mortality of elderly people in nursing homes due to COVID-19. Similarly, given the limitations of the study, it may be the case that there are other variables that influence cognitive impairment, such as central obesity or vascular pathology. These aspects were not considered due to the cross-sectional nature of the study and the lack of knowledge during the early stages of the pandemic. Finally, it is important to note that our study does not include a comparative group of isolated geriatric patients without pathology. This is because, except for two patients, the rest contracted COVID-19. Additionally, there is a possibility that cognitive deterioration may be due to the natural deterioration process in a vulnerable population.

## Conclusions

The findings of this research indicate a significant reduction in the cognitive abilities of older individuals residing in nursing homes after suffering from COVID-19 disease. Such outcomes call for action to mitigate the negative effects of social isolation on the general health and cognitive decline of older people. It is essential that a multidisciplinary team assesses the social isolation of older adults to develop a strategy to prevent isolation while preserving their cognitive function, a measure that is critical to maintaining their physical and mental health especially considering the effects of COVID-19.

## Data Availability

The data in this paper are available upon a reasonable request to the corresponding author.
